# Supplementary material for: What Makes GPCRs from Different Families Bind to the Same Ligand?
Source: Biomolecules. 2022 Jun 21;12(7):863. doi: 10.3390/biom12070863 (PMC9313020; doi:10.3390/biom12070863)
Supplement: Supplementary file 1 [file biomolecules-12-00863-s001.zip › biomolecules-1749213-supplementary.pdf]

Supplementary Document

Table S1. Frequency distribution of ligands by the number of distinct families they bind to.

Number of distinct IUPHAR families	Frequency
1	98750
2	16191
3	11

Table S2. List of the 11 ligands that bind to 3 different IUPHAR families.

Ligands InChIKey[a]	Abbreviation	Compound Class
XLWJPQQFJNGUPA-UHFFFAOYSA-N	XLWJ	Synthetic Organic
DTZDSNQYNPNCPK-UHFFFAOYSA-N	DTZD	Synthetic Organic
CLQVVBPDAXJGBV-UHFFFAOYSA-N	CLQV	Synthetic Organic
AJLFQFYMLRXVHV-UHFFFAOYSA-N	AJLF	Not known
IKSHHOBCJKJKOG-UHFFFAOYSA-N	IKSH	Not known
FQUAFMNPXPXOJE-UHFFFAOYSA-N*	FQUA	Not known
MLQFOEOUNIRULR-UHFFFAOYSA-N*	MLQF	Not known
YKMSTUDOGGAEJH-UHFFFAOYSA-N	YKMS	Not known
USZPQRMQYJIDII-UHFFFAOYSA-N	USZP	Not known
BYBLEWFAAKGYCD-UHFFFAOYSA-N*	BYBL	Not known
NKOPNLUYOHOGFZ-UHFFFAOYSA-N	NKOP	Not known

[a]InChIKey: International Chemical Identifier compact hashed code

*Were excluded from further analysis

Table S3. List of ligands and the proteins they bind to for the control dataset.

Ligands PDB ID	Proteins UniProt ID	Protein PDB ID	GPCR	GPCR Class
0HK	P08173	5DSG	Yes	A
	P11229	5CXV	Yes	A
7LD	P28223	6WGT	Yes	A
	P41595	5TVN	Yes	A
7MA	O43613	6TOD	Yes	A
	O43614	5WQC	Yes	A
8NU	P14416	6CM4	Yes	A
	P28223	6A93	Yes	A
40F	Q14416	4XAQ	Yes	C
	Q14832	4XAR	Yes	C
89F	P28222	5V54	Yes	A
	P28223	6WH4	Yes	A
ADN	P29274	2YDO	Yes	A
	P30542	6D9H	Yes	A
GGL	O00222	6BSZ	Yes	C
	Q14416	5CNI	Yes	C
	Q14832	5CNK	Yes	C
GLU	A0A173M0G2	5X2P	Yes	TR2
	E9P5T5	4IO2	No	-
	P41594	3LMK	Yes	A

	P42264	3S9E	No	-
	Q14416	7MTR	Yes	A
SRO	P08908	7E2Y	Yes	A
	P37231	3ADV	No	-
97V*	P43220	5VEX	Yes	B
	P47871	5XF1	Yes	B
Z99	P41594	7FD9	Yes	C
	Q13255	3KS9	Yes	C
	Q14831	3MQ4	Yes	C
	Q14832	7WI6	Yes	C

*Predicted pockets did not include the pocket for ligand, as a result was excluded from further analysis.
